# A study to investigate the prevalence of headache disorders and migraine among people registered in a health insurance association in Japan

**DOI:** 10.1186/s10194-022-01439-3

**Published:** 2022-06-23

**Authors:** Fumihiko Sakai, Koichi Hirata, Hisaka Igarashi, Takao Takeshima, Takeo Nakayama, Hiromi Sano, Hiroyuki Kondo, Yoshiyuki Shibasaki, Nobuyuki Koga

**Affiliations:** 1 Saitama International Headache Center, Saitama Neuropsychiatric Institute, 6-11-1 Honmachi-Higashi, Chuo-ku, Saitama, 338-8577 Japan; 2 Department of Neurology, Dokkyo Medical University, 880 Kitakobayashi, Mibu, Shimotsuga, Tochigi 321-0293 Japan; 3 Department of Internal Medicine, Headache Care Unit, Fujitsu Clinic, 4-1-1 Kamikodanaka, Nakahara-ku, Kawasaki, Kanagawa 211-8588 Japan; 4 Headache Center, Department of Neurology, Tominaga Hospital, 1-4-48 Minatomachi, Naniwa-ku, Osaka-shi, Osaka 556-0017 Japan; 5 Department of Health Informatics, Graduate School of Medicine and School of Public Health, Kyoto University, Yoshida-Konoe-cho, Sakyo-ku, Kyoto, 606-8501 Japan; 6 Medical Affairs, Otsuka Pharmaceutical Co., Ltd, 3-2-27 Otedori, Chuo-ku, Osaka, 540-0021 Japan; 7 Medical Affairs, Otsuka Pharmaceutical Co., Ltd., Shinagawa Grand Central Tower, 2-16-4, Konan, Minato-ku, Tokyo, 108-8242 Japan; 8 Medical Affairs, Otsuka Pharmaceutical Co., Ltd., 463-10 Kagasuno, Kawauchi-cho, Tokushima, 771-0192 Japan

**Keywords:** Migraine prevalence, Headaches, Database study, Online survey, Medical claims, Japan, MS-QOL, WPAI

## Abstract

**Background:**

Migraine is a chronic disease characterized by episodic headache attacks. No recent studies have, however been conducted on the epidemiology of migraine or the treatment landscape in Japan. This study was conducted as a fact-finding survey using medical claims data and an online survey on migraine and headaches, conducted among members of health insurance associations with the objective of gaining an understanding of migraine prevalence and the treatment status in Japan.

**Methods:**

The study methodology utilized a unique approach of combined data sources. The data sources used in this study are medical claims data and linked online survey data provided by DeSC Healthcare Inc (DeSC). The primary outcomes (from survey responses) were: the overall number and proportion of migraine patients; and the overall prevalence of migraine, stratified by age and sex. The secondary outcomes (from survey responses) were use of medical care; and clinical features/headache symptoms. The analysis population included all individuals who had response data for surveys conducted by DeSC. The online survey data and medical claims data were summarized.

**Results:**

The data population comprised 21,480 individuals. On the basis of the survey results, including probable cases, the overall prevalence of migraine was 3.2%. The highest prevalence of migraine was observed in patients aged 30–39 years. The prevalence of migraine in women was 4.4 times higher than in men. The percentage of migraine patients who had not been seen by a doctor was 81.0%. More than 80% of patients were taking over-the-counter drugs and 4.8% took prescription medicines only. Approximately 52.9% of patients considered that the intensity of pain symptoms was severe. Moreover, the majority of patients (72.9%) considered that the impairment of daily life activities was of moderate or severe degree.

**Conclusions:**

In Japan, the percentage of people with migraine who did not receive medical attention is as high as 80%. Additionally, the majority of patients tend to endure symptoms and continue with everyday activities. With innovative treatment approaches becoming available it is necessary to disseminate information that migraine is not a simple headache but an illness that requires medical treatment and consultation.

**Supplementary Information:**

The online version contains supplementary material available at 10.1186/s10194-022-01439-3.

## Background

Migraine is a chronic disease characterized by episodic headache attacks. The headache attacks are described as unilateral and pulsating, of moderate to severe intensity, and lasting 4–72 h [1]. In addition to “headache”, migraine may also be accompanied by symptoms such as nausea and vomiting, photophobia, phonophobia, and osmophobia, as well as visual abnormalities, including aura (jagged geometric patterns of glittering lights that gradually obscure vision, mosaic of parts of vision, etc.), which greatly interfere with daily life [2].

There are nine reports of epidemiological studies of headache disorders in Japan, [3–11], of which six are in adults [4, 7–11]. According to a nationwide telephone survey by Sakai & Igarashi reported in 1997 [3], the prevalence of tension-type headache was 15.6% and that of migraine was 8.4%. The prevalence of migraine by sex was higher in females (12.9% compared to 3.6% in males), with the highest incidence in the 30–40 age group. The results also showed that 74% of people that experienced migraine symptoms reported that their headache attacks interfered with their daily lives, but they were generally able to conduct work and other social activities. The percentage of those who had never been to a medical institution for migraine was 69.4%. In terms of medication status, only 5.4% of patients took prescription medication, and 56.8% used over-the-counter (OTC) analgesics only. Overall, these reports showed that many people with migraine do not seek medical attention; rely on OTC analgesics for headache treatment; and do not take time off from social activities (or endure them) even when headaches interfere with daily life. This situation has been described as Japanese migraine patients “suffering in silence [12]”.

In the field of headache medicine, triptans, which are serotonin 1B/1D receptor agonists, were approved in Japan in 2001 for the acute treatment of migraine. Until then, analgesia with non-steroidal anti-inflammatory drugs (NSAIDs) had been the mainstay of migraine treatment, but the introduction of a mechanism-of-action treatment that matched the migraine onset mechanism was expected to improve treatment effectiveness by reducing the number of headache attacks and attack severity. In addition, it was expected that use of triptans would increase the number of people who visited medical institutions rather than enduring headaches. The 2021 Guidelines for the Management of Chronic Headache placed triptans as the first-line treatment for the acute phase of moderate-to-severe migraine headache [1], which was a change in migraine treatment policy. Due to the fact that no actual research studies evaluating triptans have been reported, it is still unclear, however, how the low consultation rate; reliance on OTC analgesics; changes in disruption of daily life, quality of life; and consultation trends have changed due to the use of triptans.

In 2019 monoclonal antibodies drugs targeting the calcitonin gene-related peptide (CGRP) or the CGRP receptor with a novel mechanism of action for migraine were launched in the United States and Europe. CGRP is a neuropeptide that was first reported to be associated with migraine in 1982, and subsequent studies have also shown an association with migraine. In Japan, monoclonal antibodies drugs targeting CGRP or its receptor are approved, or under evaluation in current clinical trials, and are expected to provide further improvement in migraine medication.

Two studies, on the actual condition of migraine were reported in 2019: an analysis of disease burden and treatment patterns in migraine patients using the Adelphi Migraine Disease Identification Program (a survey developed in the UK) [7], and an analysis of drug dosing patterns for migraine medications using the Japan Medical Data Center (JMDC) (medical claims database) database [8]. These reports are based on surveys of migraine patients and the analysis of prescription patterns using claims data. Due to the limits of this approach the current status in Japan, however, is that the actual prevalence of migraine is not clear. Additionally, it is not clear how the migraine sex ratio, migraine severity, migraine impact on daily life, medication status, and consultation trends in the general population, which were reported about 20 years ago [3], have changed since the launch of triptans.

The main purpose of this study was to gain an understanding of the actual prevalence of migraine and the treatment status in Japan. Additionally, the results of this study help clarify the current status of medical consultation and the reasons for discontinuing existing preventive medications.

## Methods

### Study design and data source

The study was conducted as a fact-finding survey using the unique combination of medical claims data from a health insurance association (HIA) and the linked results of an online survey on migraine and headaches conducted among members of health insurance associations contracted by DeSC Healthcare Inc (DeSC)., and individuals registered with the health promotion support service computer application, known as kencom, provided by DeSC. This combined use of data sources is thought to be unique and a particular strength of this study design.

**Fig. 1:**
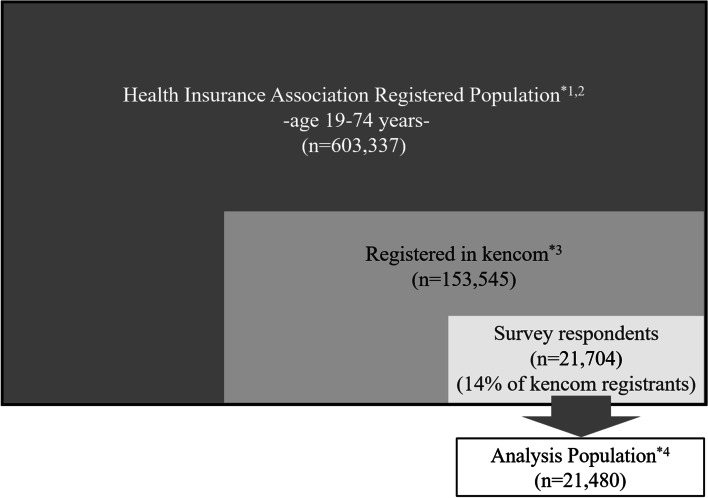
Description of the study population. ^*^1 Insured: Insured persons and their dependents. ^*^2 Persons in the population that have registered information and claims information if they have visited medical institution. ^*^3 Survey responses, claims information, and registration information can be merged with registered ID. ^*^4 Among 21,704 survey respondents, 21,480 people were included in the analysis population. A total of 224 respondents were excluded from the analysis population as their age and gender did not match the claims data

Figure 1 provides a description of the study population. Medical claims data was acquired from the “Health Insurance Association Registered Population”. This association comprises individuals aged 19–74 years and is managed by DeSC. Individuals registered in the health insurance association are offered the opportunity to voluntarily enroll in a health promotion support service computer application, known as kencom. In the conduct of this study individuals registered with kencom were invited to participate in on-line health surveys. The health survey was conducted over a one month period of 1^st^ to 30^th^ November 2020, with a month response period. To avoid potential bias no mention of migraine was made in the health survey invitations. The individuals from whom health surveys were received comprised the survey population. The data sources used in this study are, therefore, medical claims data and online survey data. This data source is anonymously processed information comprising medical claims and survey data provided by the Society-Managed Employment-Based Health Insurance prior to the start of this study. Survey data was anonymously processed after data linkage with the medical claims data based on individual consent. Therefore, the research that was conducted in this study used only anonymously processed information that had already been created.

The HIA database contains individual-level demographic information (e.g., age, sex) and dated medical information for inpatient and outpatient service provided by health care organizations and pharmacies (e.g., start of treatment, name of procedure, name of prescription and disease coded in the International Statistical Classification of Diseases and Related Health Problems, 10th revision, name of medical service, cost,). Provided that the employee did not leave the insurance program (e.g. retiring, changing jobs), it was possible to trace the medical and treatment history from multiple institutions.

### Ethics statement

As this survey used only anonymized data, and because Otsuka Pharmaceutical Co., Ltd., Clinical Study Support Inc. (CSS)., and the medical experts did not possess or receive data correspondence sheets, it was impossible to identify any individual. In addition, DeSC does not have a correspondence table for the data provided to Otsuka Pharmaceutical Co., Ltd, and it is, therefore, impossible to identify individuals from this data. Therefore, no new individual level consent was obtained for the use of the data in this study. However, the study protocol was approved by the ethics committee of the Research Institute of Healthcare Data Science (approval No.: RI2020012). Additionally, this survey was, however, conducted in consideration of the Declaration of Helsinki (revised October 2013) by the World Medical Association and the Ethical Guidelines for Medical Research Involving Human Subjects.

### Study population

We extracted online survey response data and medical claims data from the database for the past three years including the month in which the survey was conducted from 1 December 2017 to 30 November 2020. The study population included in the study was defined as “all individuals whose response data were available”.

### Outcome measures

#### Primary outcomes

The primary outcomes (based on the online survey response) were:

1. The overall number and proportion of people with migraine.
2. The overall prevalence of migraine, stratified by age and sex.

The definition of migraine (Additional file 1) was based on the structured survey response, including internal diagnostic criteria, as migraine with and without aura, including probable migraine according to the International Classification of Headache Disorders, 3rd edition (ICHD-3) [13]. Additionally, prevalence of tension-type headache and cluster headache which were also classified according to ICHD-3 were also examined. Individuals not classified in any of these headache types are included in other headache types (Additional file 2).

#### Secondary outcomes

##### Use of medical care (based on the online survey response)

The use of medical care was categorized as follows: The number of individuals with migraine who made hospital visits for headache (regularly visited, not regularly visited, not visited) within 6 months before answering the survey, overall and stratified by age and sex; frequency of hospital visits (< once/month, once/month, once/2 months, once/3 months, < once/3 months); reasons for visiting a hospital (unable to tolerate headaches, worried about other brain diseases, increased headache frequency, and OTC drugs no longer effective; and reasons for not visiting a doctor OTC drugs effective, used to having headache, spontaneously resolving after endurance, or pain not sufficiently severe).

##### Clinical features and symptoms of headache (based on the online survey response)

Clinical features/symptoms were classified as follows: symptoms of headache (nausea or vomiting, stiff shoulder, and neck pain); site of pain (unilateral, bilateral, frontal, occipital, periorbital, other locations); time of day of headache onset (Upon waking, morning, afternoon, evening, other, no particular time of day); headache triggers (fatigue physical or mental stress, severe weather systems such as typhoons, lack of sleep, turning points of the seasons, sunny or rainy days, work or housework, and menstruation); activities that were refrained from or reduced by headache (Operating a computer or smart phone, drinking alcohol, exercising such as playing sports or walking, going to crowded places, driving a car, housework (excluding grocery shopping, laundry, and cooking), cooking, socializing with friends and playing with children, going grocery shopping, and taking public transportation).

##### Medication use (based on the online survey response and medical claims data) and comorbidity (medical claims data)

Medical use was classified as follows: current medication use (OTC and prescription drugs, prescription drugs only (both acute and prophylactic medications), OTC drugs only, and none); number of OTC drug class use (1 or ≥ 2 types); prescription drugs for prophylactic treatments (antidepressants, anti-epileptics, calcium channel blockers, angiotensin-receptor blockers/ angiotensin converting enzyme inhibitors, Beta blockers, and others); prescription drugs for acute treatments (acetaminophen, NSAIDs, triptans, ergotamine, and antiemetic drugs); and comorbidity (hypertension, cardiovascular disease, cerebrovascular disease, gastrointestinal disorder, psychiatric and psychosomatic disorder, depression, epilepsy, asthma, allergy, and autoimmune disorder).

##### Activity impairment, MS-QOL, and Work Productivity and Activity Impairment WPAI score (based on the online survey response)

Activity impairment was classified as follows: severity of migraine when taking medicines or not taking medicines (severe, moderate, mild); impairment in daily activities (severe, moderate, mild); Migraine-Specific Quality of Life (MS-QOL) estimated using the MSQ version 2.1, which is a 14-item questionnaire measuring the impact of migraine across 3 domains during the past 4 weeks: role function-restrictive (RR) that measures functional limitations on daily, work, and social activities (7 items); role function-preventive (RP) that measures functional prevention on daily, work, and social activities (4 items); and emotional function (EF) that measures the impact on emotion (3 items) [14–16]. The source data responses were scaled to range from 0 to 100; the higher score indicating better quality of life. Work Productivity Activity Impairment (WPAI) scores were estimated using the WPAI Questionnaire-General Health for the last 7 days before questionnaire response as follows: 1) percentage of work time missed in the last week due to health conditions (absenteeism); 2) percentage of impairment while working due to health conditions (presenteeism); 3) percentage of overall work impairment due to health conditions; and 4) percentage of activity impairment due to health conditions [17].

##### Sex and age distribution of each headache type and the prevalence of headache type after weighting by age and sex (Additional file 3)

The proportion of each headache type in the analysis population was used to estimate the number of cases of each headache type among kencom registrants and health insurance members by sex and age categories. The number of cases of each headache type among kencom registrants and health insurance members by sex and age category was summed for each headache population, from which the prevalence rate was calculated.

### Statistical analysis

The analysis population included all individuals who had response data for the surveys conducted by DeSC. Demographic and clinical characteristics were descriptively summarized for overall individuals with migraine. For continuous variables, the mean ± standard deviation and median (minimum, maximum) were presented. For categorical variables, the number and percentage were presented. Post-hoc analysis of other headaches was conducted using criteria from the ID-Migraine [18] and the 4-item simple migraine screener [19]. Additionally, a sensitivity analysis of the health outcomes of kencom users was conducted, to evaluate whether kencom users showed greater health conscious behaviors than other patients. All statistical analyses were performed in SAS Release 9.4 (SAS Institute, Inc., NC, USA).

## Results

### Prevalence

**Fig. 2:**
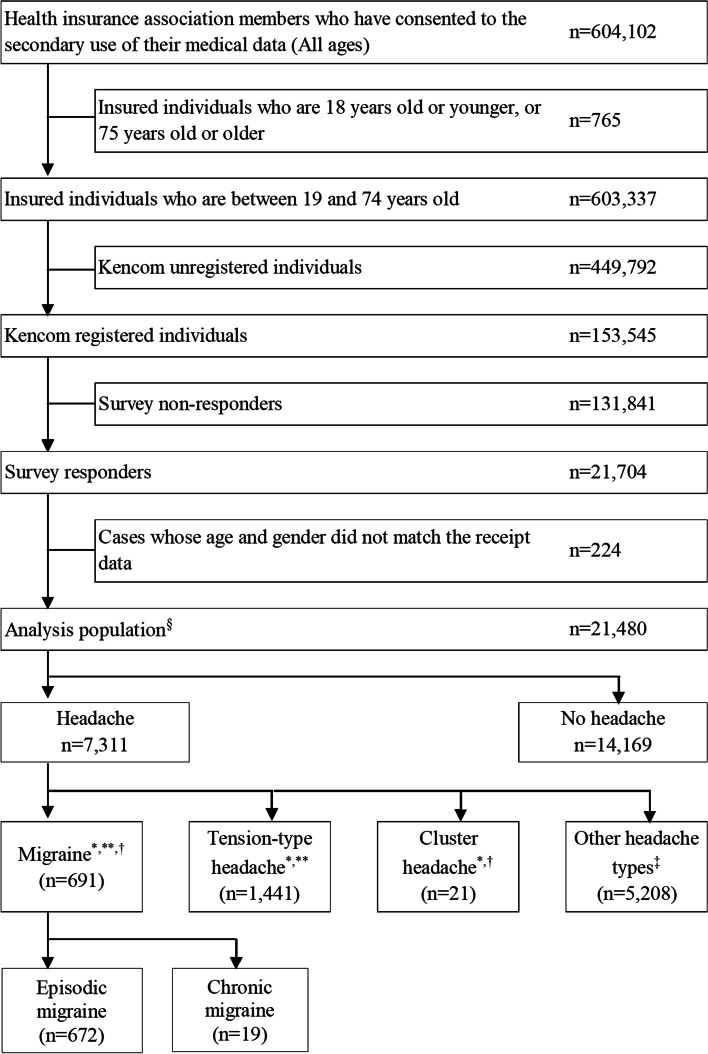
Patient disposition. ^*^ Each group except the group of other headache types included individuals classified as probable migraine, probable tension-type headache, or probable cluster headache, respectively. ^**^ There were 42 individuals who were classified into both migraine and tension-type headache. ^†^ There were 8 individuals who were classified into both migraine and cluster headache. ^‡^ Post-hoc analysis of the “other headache types” showed 261 people who had two or more matches with ID Migraine [18] and 286 people who had two or more matches with the 4-item simple migraine screener [19]. § Among the analysis population (*n* = 21,480), 7.1% of the individuals included in the analysis set were dependents of individuals directly insured by the health insurance association

The study flow is displayed in Fig. 2. A total of 603,337 individuals were registered in the health insurance association. Medical claims health data were available from individuals in this population that had visited a medical institution during the study period. From the health insurance association registered population, a total of 153,545 individuals had chosen to register in the kencom health promotion support service. Amongst individuals registered with kencom, 21,480 individuals completed the online surveys and comprised the study population.

**Fig. 3:**
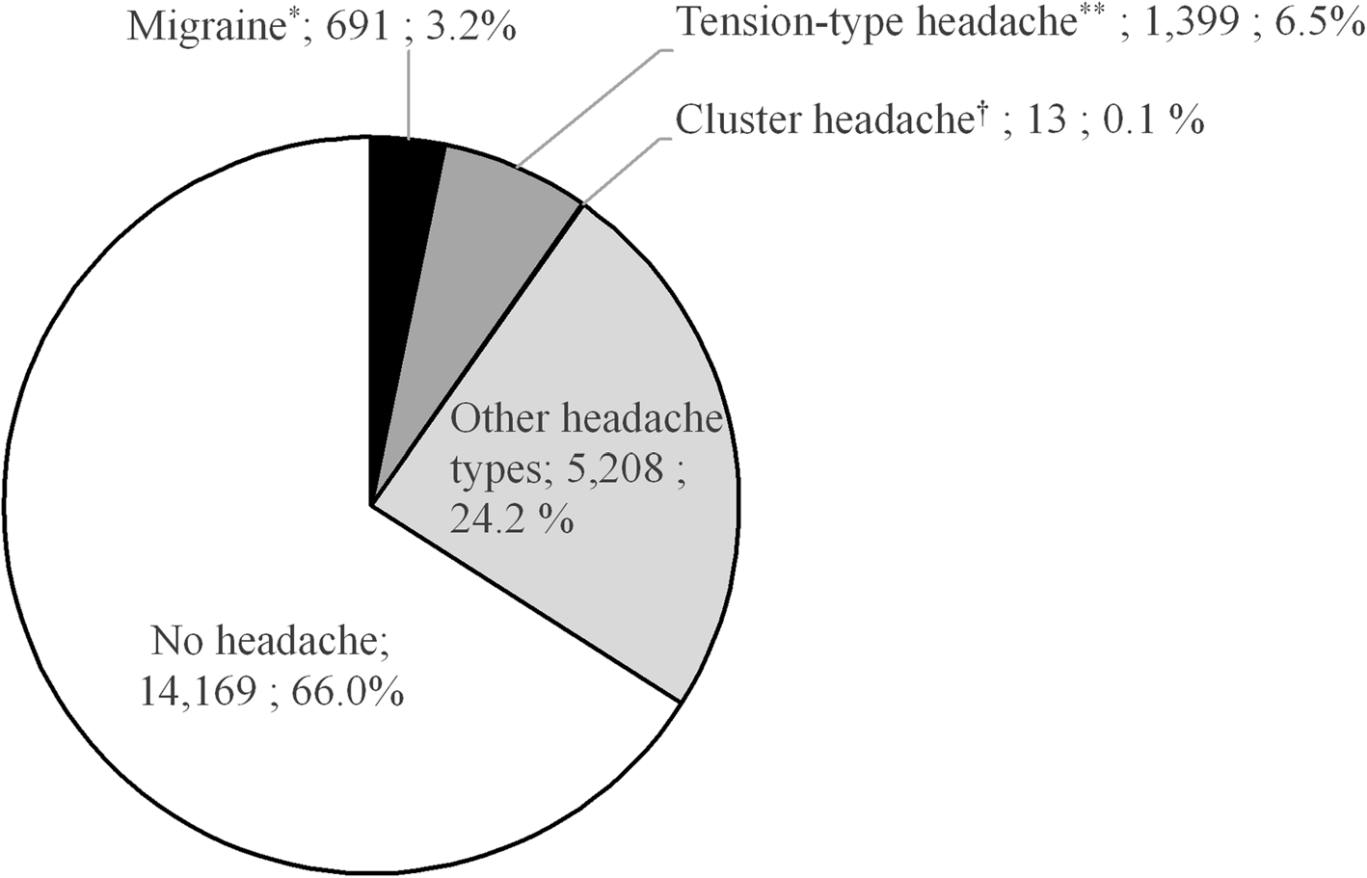
Prevalence of headaches. ^*^ The prevalence of migraine (including probable migraine) is as shown in the figure above. ^**^ The prevalence of tension-type headache (including probable tension-type headache) was 6.7% (1441 patients), including concomitant probable migraine (3 patients), concomitant migraine (3 patients), and probable migraine (36 patients). ^†^ The prevalence of cluster headache was 0.1% (21 patients), including concomitant migraine (2 patients) and probable migraine (6 patients)

On the basis of the online survey results, that included probable cases, the overall prevalence of migraine was 3.2% (691/21,480) (Fig. 3). After weighting the data by age and sex among kencom registrants (Additional file 3) the prevalence of migraine was 3.5%. Whereas, after weighing the data by sex and age among health insurance association members (Additional file 3), the prevalence of migraine was 4.2%. From medical claims data (Table 1) the prevalence of migraine was 1.0% (208/21,480).

**Table 1:** Characteristics of people with migraine

Variables		Analysis population (*N* = 21,480)		Migraine^a^ (*N* = 691)							
				Migraine total (*N* = 691)			Chronic headache (*N* = 19)			Episodic headache (*N* = 672)	
		n	%	n		%	n	%		n	%
Sex	Male	15,802	73.6		272	39.4	5		26.3	267	39.7
	Female	5,678	26.4		419	60.6	14		73.7	405	60.3
Age, Total	19–29 years	1,151	5.4		60	8.7	1		5.3	59	8.8
	30–39 years	2,944	13.7		184	26.6	7		36.8	177	26.3
	40–49 years	6,095	28.4		262	37.9	7		36.8	255	37.9
	50–59 years	8,265	38.5		175	25.3	4		21.1	171	25.4
	≥ 60 years	3,025	14.1		10	1.4	0		0.0	10	1.5
Male	19–29 years	675	3.1		19	7.0	0		0.0	19	7.1
	30–39 years	1,921	8.9		84	30.9	3		60.0	81	30.3
	40–49 years	4,035	18.8		94	34.6	2		40.0	92	34.5
	50–59 years	6,483	30.2		71	26.1	0		0.0	71	26.6
	≥ 60 years	2,688	12.5		4	1.5	0		0.0	4	1.5
Female	19–29 years	476	2.2		41	9.8	1		7.1	40	9.9
	30–39 years	1,023	4.8		100	23.9	4		28.6	96	23.7
	40–49 years	2,060	9.6		168	40.1	5		35.7	163	40.2
	50–59 years	1,782	8.3		104	24.8	4		28.6	100	24.7
	≥ 60 years	337	1.6		6	1.4	0		0.0	6	1.5
Years lived with headache	-5 years	-	-		57	8.2	2		10.5	55	8.2
	6—10 years	-	-		54	7.8	3		15.8	51	7.6
	11—15 years	-	-		50	7.2	2		10.5	48	7.1
	16—20 years	-	-		57	8.2	2		10.5	55	8.2
	21—25 years	-	-		49	7.1	1		5.3	48	7.1
	26—30 years	-	-		34	4.9	2		10.5	32	4.8
	≥ 31 years	-	-		62	9.0	4		21.1	58	8.6
Area of residence	Hokkaido	-	-		6	0.9	0		0.0	6	0.9
	Tohoku	-	-		23	3.3	0		0.0	23	3.4
	Kanto-Koshinetsu	-	-		399	57.7	9		47.4	390	58.0
	Hokuriku	-	-		8	1.2	0		0.0	8	1.2
	Chubu	-	-		86	12.4	4		21.1	82	12.2
	Kinki	-	-		121	17.5	4		21.1	117	17.4
	Chugoku	-	-		10	1.4	1		5.3	9	1.3
	Shikoku	-	-		10	1.4	0		0.0	10	1.5
	Kyushu	-	-		28	4.1	1		5.3	27	4.0
Job category^b^	Administrative positions	-	-		231	33.4	8		42.1	223	33.2
	Professional and technical personnel	-	-		186	26.9	5		26.3	181	26.9
	Housewife (husband)	-	-		58	8.4	2		10.5	56	8.3
	Managers	-	-		47	6.8	0		0.0	47	7.0
	Others	-	-		169	24.5	4		21.1	165	24.6
Annual household income (including tax)	< 1,000,000 JPY (< 9,000 USD^c^)	-	-		11	1.6	1		5.3	10	1.5
	≥ 1,000,000 to < 5,000,000 JPY (≥ 9,000 to < 44,000 USD^c^)	-	-		136	19.7	4		21.1	132	19.6
	≥ 5,000,000 to < 10,000,000 JPY (≥ 44,000 to < 87,000 USD^c^)	-	-		360	52.1	10		52.6	350	52.1
	≥ 10,000,000 JPY (≥ 87,000 USD^c^)	-	-		119	17.2	2		10.5	117	17.4
	Don't know	-	-		54	7.8	2		10.5	52	7.7
	No reply	-	-		11	1.6	0		0.0	11	1.6
Aura	With aura	-	-		230	33.3	-		-	-	-
	Male	-	-		89	38.7	-		-	-	-
	Female	-	-		141	61.3	-		-	-	-
	Without aura	-	-		461	66.7	-		-	-	-
	Male	-	-		183	39.7	-		-	-	-
	Female	-	-		278	60.3	-		-	-	-
Number of days with a headache in the past 3 months	n	-	-		691	100.0	19		100.0	672	100.0
	Mean	-	-		11.1		60.5			9.7	
	SD	-	-		12.3		14.6			8.9	
	Min	-	-		1.0		45.0			1.0	
	Median	-	-		7.0		60.0			7.0	
	Max	-	-		90.0		90.0			60.0	
Number of days with a headache in the past 30 days	n	-	-		691	100.0	19		100.0	672	100.0
	Mean	-	-		5		19			4	
	SD	-	-		5.0		5.1			4.3	
	Min	-	-		0.0		15.0			0.0	
	Median	-	-		3.0		20.0			3.0	
	Max	-	-		30.0		30.0			30.0	
Receipt code for migraine in the past 6 months^d^	Yes	-	-		61	8.8	-		-	-	-
Diagnosed with migraine at the medical institution	Yes	-	-		167	24.2	6		31.6	161	24.0

*Abbreviations*: *JPY* Japanese yen, *USD*^b^, United States dollar, *SD* Standard deviation, *min* Minimum, *max* Maximum

^a^ Migraine included individuals classified as probable migraine

^b^ Details are listed in Additional File 1

^c^ USD was estimated based on the exchange rate of 1 JPY = 0.0087 USD on 09 February 2022

^d^ Data were derived from the medical claims database

Of those individuals classified as having migraine in the survey, 8.8% (61/691) had a migraine diagnosis in their medical claims data (Table 1). Among this population of people with migraine that were identified in both the survey and in the medical claims data, the prevalence of chronic migraine was 2.7% (19/691) (Fig. 2).

Prevalence of other headache types are shown in Fig. 3. Among individuals that had "other headache types", 261 patients had at least two matches for ID-Migraine and 286 patients had at least two matches in the 4-item-simple migraine screener (Fig. 2).

**Fig. 4:**
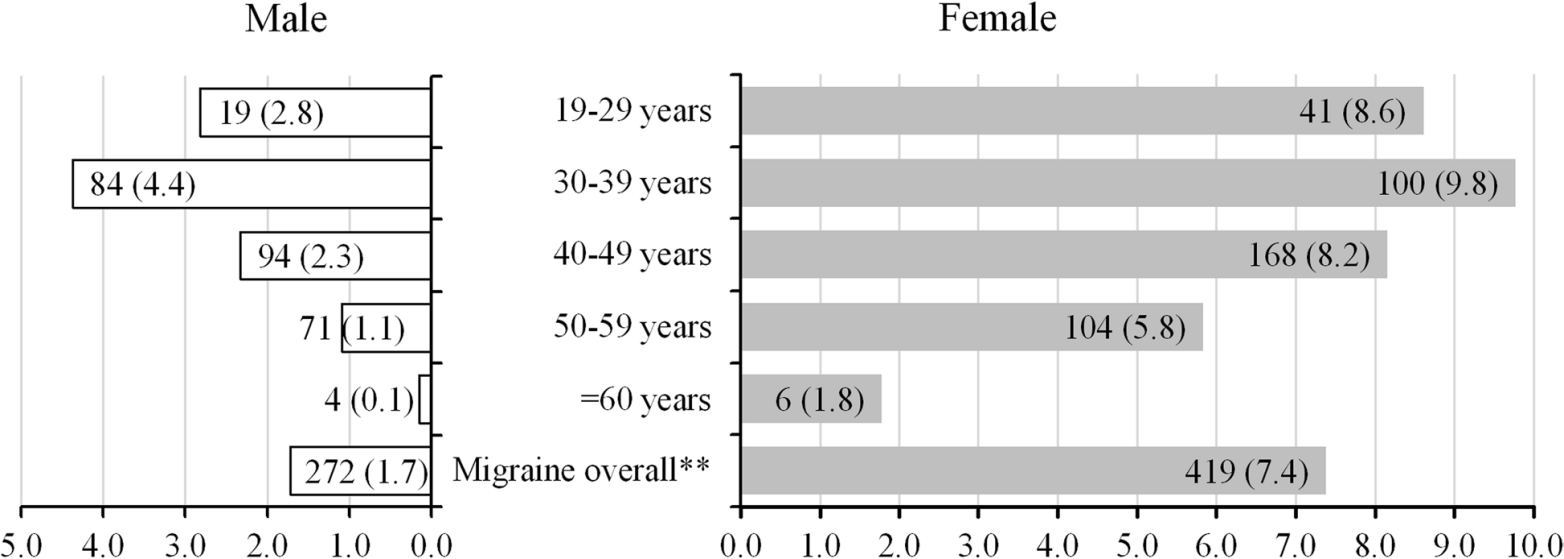
Prevalence^*^ of migraine stratified by sex and age†. ^*^ Number and percentage with the number of same age and sex category was used as a denominator. ^**^Number of migraine overall in male is 272 and female is 419. ^†^Refer the summary of number of patients for each sex in Table 1

By age, among people that experienced migraine, the highest proportion was observed in 37.9% (262/691) of patients aged 40–49 years; followed by 26.6% (184/691) of patients aged 30–39 years; and 25.3% (175/691) of patients aged 50–59 years (Table 1). Additionally Fig. 4 shows the prevalence of migraine stratified by age and sex, with the number of same age and sex category used as the denominator. After stratifying by age and sex, the prevalence of migraine in men and women aged 30–39 years was 4.4% and 9.8% respectively, showing that individuals in the range 30–39 are most likely to experience migraine headache.

Regarding sex, the prevalence of migraine in women was 4.4 times higher than in men (men: women = 1.7%: 7.4%; Fig. 4). After weighting the data by sex and age among kencom registrants (Additional file 3) the ratio of women experiencing a migraine episode was 3.75 times that of men (men: women = 2.0%: 7.5%; Additional File 3). Whereas after weighting the data by sex and age among health insurance association members (Additional file 3), the ratio of women experiencing a migraine episode was 3.1 of that in men (men: women = 2.2%: 6.9%).

### Use of medical care

The percentage of people with migraine who had not visited a doctor was 81.0% (560/691) (Table 2). For people that did attend a hospital, the percentage of individuals that visited a clinic was 3.1 times that of general hospitals. The specialties of the clinics in descending order were internal medicine, neurosurgery, and neurology (including multiple responses from online survey for hospitals/clinics). For the characteristics of people with migraine by job category please refer to Additional file 4.

**Table 2:** Current medical visits for headaches in the migraine group^a^

	Regularly visited		Not regularly visited		Not visited	
	n	%	n	%	n	%
Migraine total (*N* = 691)	44	6.4	87	12.6	560	81.0
Male (*N* = 272)	14	5.1	37	13.6	221	81.3
19–29 years	0	0.0	2	0.7	17	6.3
30–39 years	0	0.0	11	4.0	73	26.8
40–49 years	7	2.6	9	3.3	78	28.7
50–59 years	7	2.6	14	5.1	50	18.4
≥ 60 years	0	0.0	1	0.4	3	1.1
Female (*N* = 419)	30	7.2	50	11.9	339	80.9
19–29 years	4	1.0	0	0.0	37	8.8
30–39 years	5	1.2	9	2.1	86	20.5
40–49 years	14	3.3	28	6.7	126	30.1
50–59 years	6	1.4	12	2.9	86	20.5
≥ 60 years	1	0.2	1	0.2	4	1.0

^a^ Migraine included individuals classified as probable migraine

The majority of people with migraine visited a doctor less than once a month (Table 3). The reasons for seeing a doctor were as follows: because they were unable to tolerate headaches (15.3%, 106/691); because they were worried about other brain diseases (9.4%, 65/691); because of increased headache frequency (8.0%, 55/691); and because OTC drugs were no longer effective (6.9%, 48/691) (Table 3).

**Table 3:** Summary of medical visits in people with migraine

Variables		Migraine^a^ (*N* = 691)	
		n	%
Frequency of medical visits (past 6 months)	> Once/week	2	0.3
	Once/2 weeks	0	0.0
	Once/month	22	3.2
	Once/2 months	10	1.4
	Once/3 months	5	0.7
	< Once/3 months	5	0.7
Reasons for initially seeing a doctor for headache^**b**^ (multiple answers)	Unable to tolerate headaches	106	15.3
	Worried about other brain diseases	65	9.4
	Increased headache frequency	55	8.0
	OTC analgesics no longer effective	48	6.9
Reasons for seeing a doctor once for headache and not seeing thereafter^b^ (multiple answers)	Relieved not to have a brain disease that threatened life	18	2.6
Reasons of not seeing a doctor in the past 3 years^b^ (multiple answers)	OTC analgesics effective	260	37.6
	Used to having a headache	155	22.4
	Spontaneously resolving after endurance	143	20.7
	Pain not sufficiently severe	114	16.5

*Abbreviations*: *OTC* Over-the-counter

^a^ Migraine included individuals classified as probable migraine

^b^ Details are listed in Additional File 1

A total of 2.6% of people with migraine (18/691) stopped attending a hospital after one visit (Table 3) and the reason provided was because they were relieved not to have a brain disease that threatened life.

Among people with migraine (Table 3) the reasons for not seeing a doctor Additional file 5) were as follows: because OTC drugs were effective (37.6%, 260/691); because they were used to having a headache (22.4%, 155/691); because the symptoms would spontaneously resolve after endurance (20.7%, 143/691); and pain not sufficiently severe (16.5%, 114/691).

### Clinical features and symptoms

The reported symptoms of headaches included nausea and vomiting (49.9%, 345/691), stiff shoulders (35.9%, 248/691), and neck pain (26.8%, 185/691) (Tables 4 and 5). The most common locations for headaches were unilateral (84.7%, 585/691) and periorbital (36.0%, 249/691).

**Table 4:** Symptoms and triggers of migraine

Variables		Migraine^a^ (*N* = 691)	
		n	%
Symptoms associated with headache^b^ (multiple answers)	Nausea or vomiting	345	49.9
	Stiff shoulder	248	35.9
	Neck pain	185	26.8
Site of pain (multiple answers)	Unilateral	585	84.7
	Bilateral	198	28.7
	Frontal	181	26.2
	Occipital	178	25.8
	Periorbital	249	36.0
	Other	17	2.5
Time of day (single answer)	Upon waking	195	28.2
	Morning	143	20.7
	Afternoon	302	43.7
	Evening	142	20.5
	Other	13	1.9
	No particular time	223	32.3
Headache triggers^b^ (multiple answers)	Fatigue	327	47.3
	Mental or physical stress	307	44.4
	Severe weather systems, such as typhoons	286	41.4
	Lack of sleep	261	37.8
	Turning points of the seasons	208	30.1
	Sunny or rainy days	191	27.6
	Work or housework	178	25.8
	Menstruation^c^	175	25.3

^a^ Migraine included individuals classified as probable migraine

^b^ Details are in Additional file 3

^c^ Among those who had menstruation (325 patients), 171 patients (52.6%) answered "menstruation"

**Table 5:** Activities refrained from to reduce the frequency of headaches in people with migraine (stratified by sex)

Variables		Migraine^a^					
		Migraine total (*N* = 691)		Male (*N* = 272)		Female (*N* = 419)	
		n	%	n	%	n	%
Activities refrained from or reduced by headache	Operating a computer or smart phone	236	34.2	89	32.7	147	35.1
	Drinking alcohol	214	31.0	88	32.4	126	30.1
	Exercising such as playing sports or walking	129	18.7	58	21.3	71	16.9
	Going to crowded places	215	31.1	58	21.3	157	37.5
	Driving a car	92	13.3	45	16.5	47	11.2
	Housework (excluding grocery shopping, laundry, and cooking)	122	17.7	31	11.4	91	21.7
	Socializing with friends and playing with children	83	12.0	25	9.2	58	13.8
	Going to grocery shopping	108	15.6	18	6.6	90	21.5
	Taking public transportation	65	9.4	14	5.1	51	12.2
	Cooking	104	15.1	11	4.0	93	22.2

^a^ Migraine included individuals classified as probable migraine

The most common triggers provided for headache onset (Table 4 and Additional file 6) was, fatigue (47.3%, 327/691); and physical or mental stress (44.4%, 307/691). Other reasons included severe weather systems such as typhoons (41.4%, 286/691); and weather related situations including; turning points in the season (30.1%, 208/691); and sunny or rainy days (27.6%, 191/691). For women with menstruation 52.6% (171/325) selected the reason for headache onset as menstruation-related. The most common problem that migraine brings to daily life is “inability to concentrate on work or study.”

Activities refrained from or reduced by migraine symptoms in men in decreasing order (Table 5), were as follows: operating a computer or smart phone (32.7%, 89/272); drinking alcohol (32.4%, 88/272); and exercising such as playing sports or walking (21.3%, 58/272). Women selected going to crowded places (37.5%, 157/419); operating a computer or smart phone (35.1%, 147/419); drinking alcohol (30.1%, 126/419); housework (excluding grocery shopping, laundry, and cooking) (21.7%, 91/419); and grocery shopping (21.5%, 90/419).

In order to improve daily life, 55.7% of the respondents felt that they would "like the headache to disappear almost completely," and 29.6% felt that they would "like it to decrease even a little.” As the level of difficulty increased, the percentage of those who felt that "even a small decrease would be enough" decreased, while the percentage of those who felt that "almost all of the headaches should disappear" increased.

### Medication use

More than 80% of people with migraine were taking OTC drugs (89.6%, 561/626) (Table 6). Only 6.1% (38/626) of patients took prescription medicines only and 31.8% (199/626) of patients took both OTC and prescription medicines. Regarding OTC drugs, 22.9% (158/691) of people with migraine took more than one type of OTC medication. Acute care prescription medications were prescribed to 34.9% (241/691) of the respondents. Acetaminophen and NSAIDs were prescribed to 29.8% (206/691) of patients; and triptans to 5.9% (41/691) of patients. Prescription drugs for prophylaxis were prescribed to 8.1% (56/691) of patients. These medications included antidepressants (3.3%, 23/691), antiepileptics (1.9%, 13/691), and calcium channel blockers (1.6%, 11/691). Whilst 59 patients were prescribed triptans for "other headaches," as triptans are only used for migraine, it is highly likely that the "other headache" category includes migraine patients.

**Table 6:** Headache medications in migraine

Variables		Migraine^a^ (*N* = 691)		
		n		%
Medication use^b^ (past 6 months)	OTC and prescription drugs^c^	199		31.8
	Prescription drugs only (acute and prophylactic)^d^	38		6.1
	OTC drugs only	362		57.8
	No prescription drugs^d^	27		4.3
Number of OTC analgesic types	1	403		58.3
	2 or more	158		22.9
Types of prescription drugs^d^ (prophylactic, past 6 months)	Total	56		8.1
	Antidepressants	23		3.3
	Anti-epileptics	13		1.9
	Calcium channel blockers	11		1.6
	ARB/ACE inhibitors	6		0.9
	Beta-blocker	4		0.6
	Others	6		0.9
Types of prescription drugs^d^ (acute, past 6 months)	Total	241		34.9
	Acetaminophen/NSAIDs	206		29.8
	Triptans	41		5.9
	Antiemetics	28		4.1
	Intravenous steroids	14		2.0
	Tranquilizer/anesthetic preparations	6		0.9
	Tramadol	1	0.1	
	Magnesium preparations	1		0.1
	Ergotamine	0		0.0
Comorbidity^d^ (past 6 months)	Hypertension	51		7.4
	Cardiovascular disorders	23		3.3
	Cerebrovascular disorders	6		0.9
	Gastrointestinal disorders	386		55.9
	Constipation	50	7.2	
	Psychiatric/Psychosomatic disorders	104		15.1
	Depression	48		6.9
	Epilepsy	6		0.9
	Asthma	51		7.4
	Allergy	123		17.8
	Autoimmune disorders	38	5.5	

*Abbreviations*: *OTC* Over-the-counter, *ARC* Angiotensin II receptor blocker, *ACE* Angiotensin converting enzyme, *NSAID* Non-Steroidal Anti-Inflammatory Drugs

^a^ Migraine included individuals classified as probable migraine

^b^ Denominator was the number who answered "yes" to a question of taking any drugs (*n* = 626)

^c^ Data used from questionnaire responses and the medical claims database

^d^ Data derived from the medical claims database

### Activity impairment, MS-QOL, and WPAI

Regarding pain intensity (Table 7), approximately 52.9% (365/691) of respondents considered that the intensity of pain symptoms was severe. Moreover, the majority of patients (72.9%) considered that the impairment of daily life activities was of moderate or severe degree.

**Table 7:** Severity of impairment associated with medication use in migraine

	Migraine^a^ (*N* = 691)			
	Not taking medicines		Taking medicines	
	n	%	n	%
Severity of migraine^b^				
Severe	365	52.9	47	6.8
Moderate	316	45.7	69	10.0
Mild	10	1.4	510	73.8
Impairment in daily activities^c^				
Severe	204	29.5	31	4.5
Moderate	300	43.4	56	8.1
Mild	187	27.1	539	78.0

^a^ Migraine included individuals classified as probable migraine

^b^ Severity of migraine was grouped into 3 level from the patients’ response for evaluating the severity of a migraine attack in 5 levels: Severe: extreme or quite a bit of pain; Moderate: moderate pain; and Mild: little pain or no pain

^c^ Impairment in daily life was also grouped into 3 level from the patients’ response for evaluating the impairment in daily life when having a migraine in 5 levels: Severe: extreme difficulty or severe disruption in daily life; Moderate: moderate difficulty in daily life; and Mild: slightly interferes with daily life or no trouble at all

**Fig. 5:**
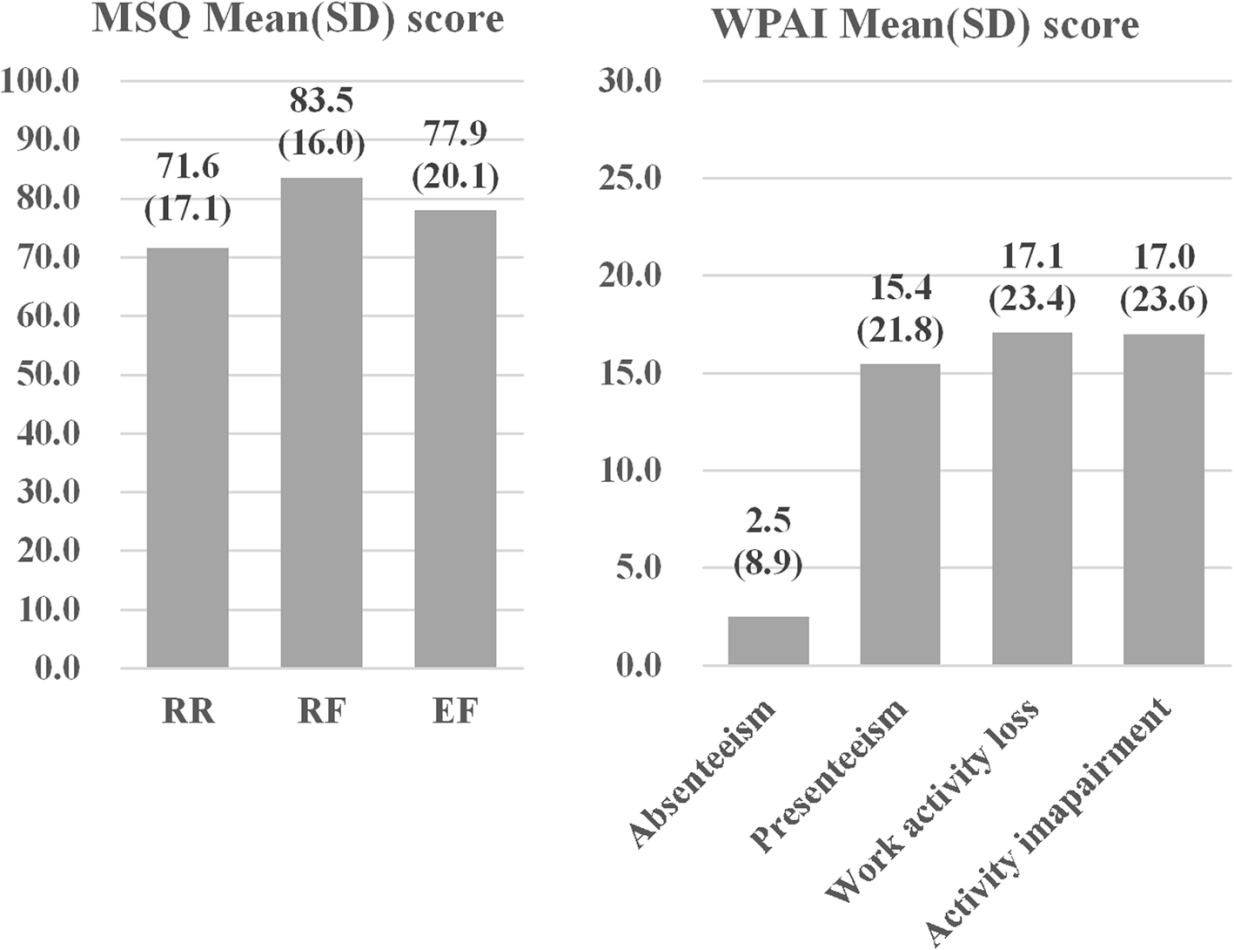
Domain scores of MSQv1.2 and WPAI in people with migraine (*n* = 691). Abbreviations: MSQ, Migraine-Specific Quality of Life Questionnaire; WPAI, Work Productivity and Activity Impairment Questionnaire; RR, role function—restrictive; RF, role function—preventive; EF, emotional function

The mean (SD) MSQ scores for migraine were 71.6 (17.1) for RR, 83.5 (16.0) for RF, and 77.9 (20.1) for EF. The mean (SD) WPAI score was 2.5% (8.9) for absenteeism, and 15.4% (21.8) for presenteeism (Fig. 5).

## Discussion

This study was conducted using a unique methodology, combing medical claims data and the results of a linked online survey on migraine and headaches, conducted among members of a health insurance association with the objective of gaining an understanding of the prevalence of migraine and the treatment status in Japan. It is the first study to investigate the prevalence of migraine in Japan for more than 20 years [3]. Whilst this is not a traditional population prevalence study, as our study population contained a large number of individuals at risk of having migraine (21,480), that was used as the denominator in calculating migraine prevalence, despite the limitations of using health insurance association data in Japan (please refer to Limitations section below) it is a valid method for investigating migraine prevalence, when it is not possible to use more traditional methods. Additionally as the response rate was relatively high, we believe that response bias is minimal.

On the basis of the survey results, including probable cases, the migraine prevalence was 3.2% (691/21,480). After weighting the data by sex and age among kencom users (Additional file 3) the prevalence of migraine was 3.5%. These migraine prevalence values were lower than the population prevalence of 8.4% reported by Sakai & Igarashi [3] in 1997. The relatively low value of migraine prevalence reported in this study’s survey results may partly be due to the fact that, whilst the questionnaires were structured, they were self-administered, and did not involve semi-structured interviews, which may have underestimated the migraine prevalence. The relative increase in migraine prevalence after weighting among kencom registrants may partially be explained by the fact that it is thought that kencom members have a higher level of health consciousness than the general Japanese population. There were, however many similar findings between our study and Sakai & Igarashi [3] that indicate that whilst migraine knowledge and treatment have progressed since 1997, many patients do not seek migraine specific treatments. These findings indicate migraine treatment and behaviors have not significantly changed since 1997.

Figure 4 shows the prevalence of migraine stratified by age and sex, with the number of same age and sex category used as the denominator. Our results are similar to previous findings [3, 4] that show migraine prevalence is higher in women than men and is most prevalent among individuals aged 30–40 years. As the kencom database includes individuals of working age, the majority of people with migraine (89.8%) were aged 30–59 years.

In our study the prevalence of migraine in women was 4.4% times higher than that in men showing that the etiology of migraine is poorly understood. This prevalence ratio among men and women remains relatively unchanged, since 1997 as Sakai & Igarashi [3] reported that the prevalence of migraine in women was 3.6 times higher than that in men.

A high percentage of people with migraine (81%) did not attend a medical institute to consult with a doctor. Consequently, as in previous studies, it is thought that the percentage of undiagnosed cases was high [3, 4]. It was also shown that people with migraine usually attempt to ameliorate pain symptoms by taking only OTC drugs (57.8%) and do not see a physician until pain becomes unbearably severe. It was also clarified that even if patients do see a physician, they will often discontinue medical institution visits if the diagnosis confirms that they don’t have a life threatening brain disease.

Regarding migraine onset, it appears there is a sex difference regarding when headaches are likely to occur. Overall, regardless of sex, the most common reason was when feeling fatigue. Additionally, there is an increased tendency for migraine onset in the following circumstances: when there are severe weather systems such as typhoons; at the turning point of seasons; and sunny or rainy days. Furthermore 52.6% of female respondents selected the reason of migraine onset to be related to menstruation, and amongst these individuals the majority endured the pain symptoms and attempted to ameliorate the pain by using OTC drugs.

Migraine symptoms were reported to cause individuals to refrain from or to reduce the conduct of everyday activities and essential household activities. Common activities included operating a computer or smartphone, drinking alcohol, and exercise such as playing sports or walking. Many women reported that they refrain from housework (excluding grocery shopping laundry and cooking) (21.7%) and grocery shopping (21.5%) due to headaches.

In assessing pain intensity, approximately 52.9% of respondents considered that the intensity of pain symptoms was severe. Moreover, the majority of patients (72.9%) considered that the impairment of daily life activities was of moderate or severe degree. This indicates that despite experiencing moderate/severe pain symptoms, the majority of patients continued with daily activities, rather than seeking medical attention or resting.

As stated above, in common with other studies [3, 4] the majority of patients used only OTC drugs (57.8%) to ameliorate pain. Only 6.1% of patients took only prescription medicines; and 31.8% of patients took both OTC and prescription medicines. Acetaminophen and NSAIDs were prescribed to 29.8% of patients; and triptans to 5.9% of patients. Prescription drugs for prophylaxis were prescribed to 8.1% of patients. Whilst 59 patients were prescribed triptans for "other headaches", as triptans are only used for migraine, it is highly likely that the "other headache" category includes migraine patients, as described below.

In our study a large number of "other headaches" was observed (24.2%). Among these "other headaches", 261 patients had two or more matches amongst three items for ID-Migraine [18] and 286 patients had two or more matches for the 4-item-simple migraine screener developed in Japan [19]. These findings suggest that a doctor's interview is important for the diagnosis of migraine. A correct migraine diagnosis, therefore, requires a detailed interview of symptoms with a doctor. This is not possible with self-administered questionnaires alone, and as it is difficult to select all patients, it is suggested that a combination of semi-structured interviews and questionnaires is useful for diagnosing migraine. Overall in our study it may be suggested that migraine headaches with symptoms of a degree of severity less than moderate were more likely to be unrecorded as migraines but as other headaches.

### Limitations of the study

This study has limitations. Our findings are not generalizable to the entire adult population with headache in Japan due to the following reasons. Firstly, as we used data from employees and their family members of large companies that are members of the health insurance association contracted by DeSC and, therefore, this is not a population prevalence study. This limited generalizability is partly mitigated by the fact that the study population, included the family members of working individuals who may not be in the workforce. It should additionally be noted, however, that self-employed persons, civil servants, employees of small and medium-sized companies, and retired elderly persons are not included. It is assumed that employees of the companies in the DeSC database have a relatively high socioeconomic status, live in areas with access to medical care, and that their occupations are limited to certain types rather than being all encompassing. Secondly, the survey was distributed only to the kencom users. The users are considered to be more health-conscious than non-users and are more likely to take positive health actions in their daily lives, which may have affected QOL and WPAI scores. Thirdly, headache types were classified according to ICHD-3 based on an online survey. As medical consultations with doctors were not conducted it was, therefore, not possible to obtain detailed information regarding symptoms. Fourthly, a large portion of our data were self-reported, and questionnaire responses are subject to recall bias. Such bias is not present in certain variables (e.g., drug prescriptions and comorbidities); however, as we used an existing medical claims database that is used for billing purposes, such data are subject to misclassification and entry error. The above mentioned limitations may have led to errors in the classification of headaches, and may in part explain why the number of cases in the “other headache types” group was large. The results of this study should be interpreted with these potential biases in mind and the possibility that individuals in the “other headache types” group may have included individuals with migraine, tension type headache, etc.

A correct migraine diagnosis, therefore, requires a detailed interview of symptoms with a doctor. This is not possible with questionnaires alone, or a combination of semi-structured interviews and questionnaires.

## Conclusion

Based on a unique method of linking medical claims and online survey data, we reported up-to-date epidemiological data of several headache disorders in Japan and showed that the current prevalence of migraine in Japan is approximately 3.2%.

In Japan, the percentage of people with migraine who did not receive medical attention is as high as 80%. Additionally, the majority of people with migraine tend to endure symptoms and continue with everyday activities. With innovative treatment approaches becoming available it is necessary to disseminate information that migraine requires specialized medical consultation and treatment. We consider that the findings of this study are of clinical value for future diagnosis and treatment of migraine headaches.

## Supplementary Information

**Additional file 1.** Definition of migraine

**Additional file 2.** ICD-10 codes for headaches and comorbidities

**Additional file 3.** Sex and age distribution of each headache type and the prevalence of headache type after weighting by age and sex

**Additional file 4.** Characteristics of people with migraine (job category)

**Additional file 5.** Summary of reasons of seeing doctors in people with migraine (*N*=691)

**Additional file 6.** Symptoms and triggers of migraine (*N*=691)

## Data Availability

The data that support the findings of this study are available from DeSC Healthcare, Inc. (Tokyo, Japan) but restrictions apply to the availability of these data, which were used under license for the current study, and so are not publicly available. Data are however available from the authors upon reasonable request and with permission of DeSC Healthcare, Inc.

## Abbreviations

OTC: Over-the-counter
NSAIDs: Non-steroidal anti-inflammatory drugs
CGRP: Calcitonin gene-related peptide
JMDC: Japan Medical Data Center
DeSC: DeSC Healthcare Inc.
CSS, Inc.: Clinical Study Support. Inc.
ICHD-3: International Classification of Headache Disorders, 3rd edition
HIA: Health Insurance Association
WPAI: Work Productivity and Activity Impairment
MS-QOL: Migraine-Specific Quality of Life
RR: Role function-restrictive
RP: Role function-preventive
EF: Emotional function
ARC: Angiotensin II receptor blocker
ACE: Angiotensin converting enzyme
MSQ: Migraine-Specific Quality of Life Questionnaire
