# Correction: A study to investigate the prevalence of headache disorders and migraine among people registered in a health insurance association in Japan

**DOI:** 10.1186/s10194-022-01529-2

**Published:** 2022-12-28

**Authors:** Fumihiko Sakai, Koichi Hirata, Hisaka Igarashi, Takao Takeshima, Takeo Nakayama, Hiromi Sano, Hiroyuki Kondo, Yoshiyuki Shibasaki, Nobuyuki Koga

**Affiliations:** 1 Saitama International Headache Center, Saitama Neuropsychiatric Institute, 6-11-1 Honmachi-Higashi, Chuo-ku, Saitama, 338-8577 Japan; 2 Department of Neurology, Dokkyo Medical University, 880 Kitakobayashi, Mibu, Shimotsuga, Tochigi, 321-0293 Japan; 3 Department of Internal Medicine, Headache Care Unit, Fujitsu Clinic, 4-1-1 Kamikodanaka, Nakahara-ku, Kawasaki, Kanagawa 211-8588 Japan; 4 Headache Center, Department of Neurology, Tominaga Hospital, 1-4-48 Minatomachi, Naniwa-ku, Osaka-shi, Osaka, 556-0017 Japan; 5 Department of Health Informatics, Graduate School of Medicine and School of Public Health, Kyoto University, Yoshida-Konoe-cho, Sakyo-ku, Kyoto, 606-8501 Japan; 6 Medical Affairs, Otsuka Pharmaceutical Co., Ltd, 3-2-27 Otedori, Chuo-ku, Osaka, 540-0021 Japan; 7 Medical Affairs, Otsuka Pharmaceutical Co., Ltd., Shinagawa Grand Central Tower, 2-16-4, Konan, Minato-ku, Tokyo, 108-8242 Japan; 8 Medical Affairs, Otsuka Pharmaceutical Co., Ltd., 463-10 Kagasuno, Kawauchi-cho, Tokushima, 771-0192 Japan

**Correction: J Headache Pain 23, 70 (2022)**

**https://doi.org/10.1186/s10194-022-01439-3**

The original publication of this article [1] contained 2 errors highlighted by the author. 1 content-related error in the abstract which needs to match the content presented in table 5, line 2: it states that the number of patients that took “Prescription drugs only (acute and prophylactic) is 6.1%.

Error 2 is another content-related error, in which, on page 4 lines 164-165 “MSQ version 2.1” needs to be changed to “MSQv1.2”.

Error 1

**The sentence currently reads:**

“…and 4.8% took prescription medicines only”

**The sentence needs to read:**

“…and 6.1% took prescription medicines only”

Error 2 (on page 4, lines 164-165)

**The sentence currently reads:**

“…Migraine-Specific Quality of Life (MS-QOL) estimated using the MSQ version 1.2…”

**The sentence needs to read:**

“…Migraine-Specific Quality of Life (MS-QOL) estimated using the MSQ version 2.1…”

Error 3:

Fig. 5 currently reads: “Domain scores of MSQv1.2....”

Fig. 5 needs to read: “Domain scores of MSQv2.1....”

## References

[1] Sakai F, Hirata K, Igarashi H, Takeshima T, Nakayama T, Sano H, Kondo H, Shibasaki Y, Koga N (2022). A study to investigate the prevalence of headache disorders and migraine among people registered in a health insurance association in Japan. J Headache Pain.

