# Supplementary material for: A study to investigate the prevalence of headache disorders and migraine among people registered in a health insurance association in Japan
Source: J Headache Pain. 2022 Jun 23;23(1):70. doi: 10.1186/s10194-022-01439-3 (PMC9219245; doi:10.1186/s10194-022-01439-3)
Supplement: Supplementary file 1 — Additional file 1. Definition of migraine [file 10194_2022_1439_MOESM1_ESM.pdf]

Additional file 1 Definition of migraine

Item*	Response	Patterns of responses
Duration (single answer)	Half a day	At least one of the “Responses” is selected
	All day	
	2 to 3 days	
Site of pain (multiple answers)	Unilateral	At least the "Response" is selected
Characteristics (multiple answers)	Throbbing or pulsating pain	At least one of the "Responses" is selected
	Pounding pain	
Change in severity due to daily activities (walking, climbing upstairs, etc.) or due to physical activity (single answer)	Worsens (avoids movement due to pain)	Either the "Response" in “Change in severity due to daily activities” or “State when in pain” is selected
State when in pain (single answer)	It is more comfortable to stay still	
Symptoms associated with headache (multiple answers)	All in the following list from	a)
	a) Nausea or vomiting	b) and c)
	b) Photophobia	a), b) and c)
	c) Phonophobia	One of the above combination is selected
Severity (single answer)	Moderate pain	At least one of the "Responses" is selected
	Quite a bit of pain	
	Extreme pain	

* If only one of the six criteria above did not apply, the patient was considered to have a "probable migraine" and was included in the migraine category.
