# Supplementary material for: A study to investigate the prevalence of headache disorders and migraine among people registered in a health insurance association in Japan
Source: J Headache Pain. 2022 Jun 23;23(1):70. doi: 10.1186/s10194-022-01439-3 (PMC9219245; doi:10.1186/s10194-022-01439-3)
Supplement: Supplementary file 2 — Additional file 2. ICD-10 codes for headaches and comorbidities [file 10194_2022_1439_MOESM2_ESM.pdf]

Additional file 2 ICD-10 codes for headaches and comorbidities

Disease category	ICD10 codes	Diseases name
Migraine	G43	Migraine
Tension type headache	G442	Tension-type headache
Cluster headache	G440	Cluster headache syndrome
Other headache types	G441	Vascular headache, not elsewhere classified
	G443	Chronic post-traumatic headache
	G444	Drug-induced headache, not elsewhere classified
	G448	Other specified headache syndromes
Epilepsy	G40	Epilepsy
	G41	Status epilepticus
Hypertension	I10-I15	Hypertensive diseases
Cardiovascular disorders	I20-I25	Ischaemic heart diseases
	I26-I28	Pulmonary heart disease and diseases of pulmonary circulation
	I30-I52	Other forms of heart disease
Cerebrovascular disorders	I60-I69	Cerebrovascular diseases
Gastrointestinal disorders	K00－K93	Diseases of the digestive system
Constipation	K590	Constipation
Psychiatric/Psychosomatic disorders	F00－F99	Mental and behavioural disorders
Depression	F32	Depressive episode
	F33	Recurrent depressive disorder
Asthma	J45	Asthma
	J46	Status asthmaticus
Allergy	J30	Vasomotor and allergic rhinitis
	L23	Allergic contact dermatitis
	J450	Predominantly allergic asthma
	K522	Allergic and dietetic gastroenteritis and colitis
	L500	Allergic urticaria
	T784	Allergy, unspecified
Autoimmune disorders	E10	Diabetes mellitus,insulin dependent
	E035, E039, E050, E055, E059, E063, E065	Autoimmune thyroid disease
	E310	Autoimmune polyglandular syndrome
	M023, M028, M029	Reactive arthritis (Reiter’s syndrome)
	M05, M06, M080, M081, M082, M083, M084	Rheumatoid arthritis
	M45	Ankylosing spondylitis

M30	Polyarteritis nodosa and related condition (Incl. Kawasaki, Churg-Strauss syndrome,etc.)
M311	Thrombotic microangiopathy
M313	Granulomatosis with polyangiitis (Wegeners's nulomatosis)
M317	Microscopic polyangiitis
D690	Henoch-Schonlein purpura
M353, M315, M316	Giant cell arteritis/ Polymyalgia rheumatica
M32	Systemic lupus erythematosus
M330, M331, M332, M339	Polymyositis/dermatomyositis
M34	Systematic sclerosis (scleroderma)
M350	Sjögren's syndrome
M351	Mixed connective tissue disease
M352	Behcet's syndrome
L100	Pemphigus vulgaris
L12	Bullous pemphigoid
L130	Dermatitis herpetiformis
L40	Psoriasis
L64	Alopecia areata
L80	Vitiligo
D510	Pernicious anemia
D590, D591	Autoimmune hemolytic anemia
D693	Idiopathic thrombocytopenic purpura
G04	Acute disseminated encephalomyelitis
G131	Anti-NMDA receptor encephalitis
G35	Multiple sclerosis
G36	Neuromyelitis optica and ADEM
G610, G611, G618, G61.9	Guillain-Barré syndrome
G700	Myasthenia gravis
K743	Primary biliary cirrhosis
K50	Crohn's disease
K51	Ulcerative colitis
K900	Coeliac disease
I00, I010, I011, I012, I018, I019, I020, I029	Acute rheumatic fever and chorea
D86	Sarcoidosis
N00, N01, N03, N05	IgA nephropaty
