# Supplementary material for: A study to investigate the prevalence of headache disorders and migraine among people registered in a health insurance association in Japan
Source: J Headache Pain. 2022 Jun 23;23(1):70. doi: 10.1186/s10194-022-01439-3 (PMC9219245; doi:10.1186/s10194-022-01439-3)
Supplement: Supplementary file 3 — Additional file 3. Sex and age distribution of each headache type and the prevalence of headache type after weighting by age and sex [file 10194_2022_1439_MOESM3_ESM.pdf]

Additional file 3 Sex and age distribution of each headache type and the prevalence of headache type after weighting by age and sex

Variables		Analysis population (N=21,480)		Registered to kencom (N=153,545)		Health insurance subscribers (N=603,337)	
		n	%	n	%	n	%
Sex and age distribution							
Male	19-29 years	675	3.1	10,820	7.0	71,154	11.8
	30-39 years	1,921	8.9	19,924	13.0	63,214	10.5
	40-49 years	4,035	18.8	30,948	20.2	75,809	12.6
	50-59 years	6,483	30.2	37,088	24.2	78,438	13.0
	≥60 years	2,688	12.5	14,848	9.7	55,312	9.2
Female	19-29 years	476	2.2	4,786	3.1	44,351	7.4
	30-39 years	1,023	4.8	8,010	5.2	43,517	7.2
	40-49 years	2,060	9.6	13,568	8.8	67,588	11.2
	50-59 years	1,782	8.3	11,018	7.2	60,749	10.1
	≥60 years	337	1.6	2,535	1.7	43,205	7.2
Prevalence of headache types after weighting by age and sex							
Overall	Migraine*	691	3.2	5,311	3.5	25,372	4.2
	Tension type headache*	1,441	6.7	10,805	7.0	46,839	7.8
	Cluster headache*	21	0.1	161	0.1	698	0.1
	Other headache types	5,208	24.2	37,990	24.7	156,290	25.9
Male	Migraine*	272	1.7	2,323	2.0	7,473	2.2
	Tension type headache*	830	5.3	6,449	5.7	20,044	5.8
	Cluster headache*	14	0.1	115	0.1	447	0.1
	Other headache types	3,198	20.2	23,926	21.1	71,217	20.7
Female	Migraine*	419	7.4	2,988	7.5	17,899	6.9
	Tension type headache*	611	10.8	4,356	10.9	26,795	10.3
	Cluster headache*	7	0.1	46	0.1	251	0.1
	Other headache types	2,010	35.4	14,064	35.2	85,073	32.8

* Each headache group included individuals classified as probable migraine, probable tension-type headache, or probable cluster headache, respectively.
