# Supplementary material for: A study to investigate the prevalence of headache disorders and migraine among people registered in a health insurance association in Japan
Source: J Headache Pain. 2022 Jun 23;23(1):70. doi: 10.1186/s10194-022-01439-3 (PMC9219245; doi:10.1186/s10194-022-01439-3)
Supplement: Supplementary file 4 — Additional file 4. Characteristics of people with migraine (job category) [file 10194_2022_1439_MOESM4_ESM.pdf]

Additional file 4 Characteristics of people with migraine (job category)

Variables		Migraine* (N=691)					
		Migraine total (N=691)		Chronic headache (N=19)		Episodic headache (N=672)	
		n	%	n	%	n	%
Job category	Administrative positions	231	33.4	8	42.1	223	33.2
	Professional and technical personnel	186	26.9	5	26.3	181	26.9
	Housewife (husband)	58	8.4	2	10.5	56	8.3
	Managers	47	6.8	0	0.0	47	7.0
	Technical and labor	41	5.9	0	0.0	41	6.1
	Part-time	41	5.9	1	5.3	40	6.0
	Sales	33	4.8	1	5.3	32	4.8
	Qualification required services (hairdresser, care worker, cook, etc.)	8	1.2	0	0.0	8	1.2
	Services without qualification	8	1.2	0	0.0	8	1.2
	Marketing	3	0.4	0	0.0	3	0.4
	Unemployed	2	0.3	0	0.0	2	0.3
	Transportation and communication	1	0.1	0	0.0	1	0.1
	Public service and maintenance	1	0.1	0	0.0	1	0.1
	Agriculture, forestry, fishery	0	0.0	0	0.0	0	0.0
	Students	0	0.0	0	0.0	0	0.0
	Other	31	4.5	2	10.5	29	4.3

* Migraine included individuals classified as probable migraine.
