# Supplementary material for: A study to investigate the prevalence of headache disorders and migraine among people registered in a health insurance association in Japan
Source: J Headache Pain. 2022 Jun 23;23(1):70. doi: 10.1186/s10194-022-01439-3 (PMC9219245; doi:10.1186/s10194-022-01439-3)
Supplement: Supplementary file 5 — Additional file 5. Summary of reasons of seeing doctors in people with migraine (N=691) [file 10194_2022_1439_MOESM5_ESM.pdf]

Additional file 5 Summary of reasons of seeing doctors in people with migraine (N=691)

a) Reason for initially seeing a doctor for headache (multiple answers)

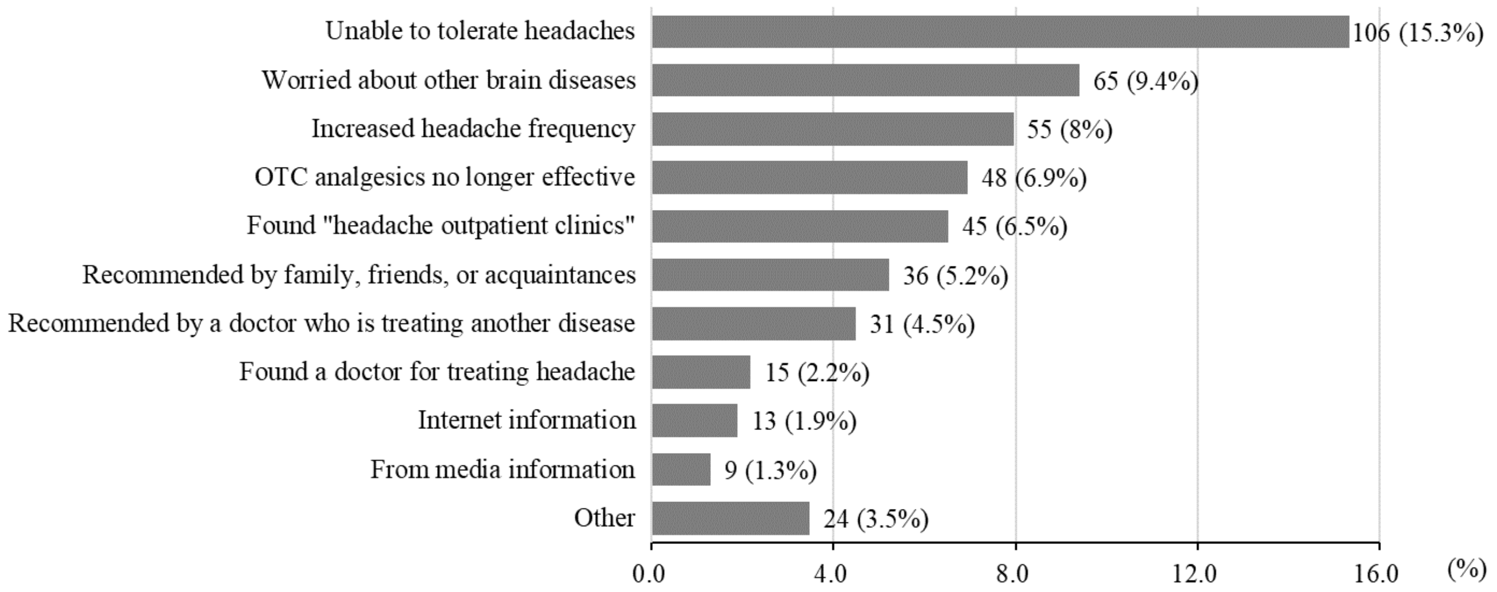

b) Reasons for seeing a doctor once for headache and not seeing thereafter (multiple answers)

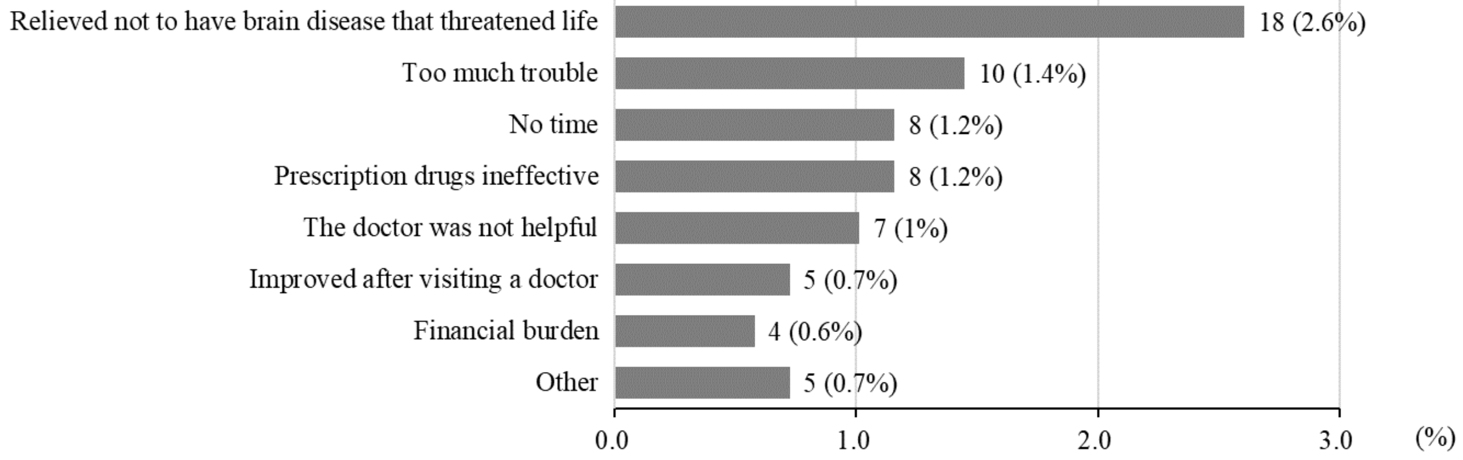

c) Reasons for not seeing a doctor for headaches in the past 3 years (multipe answers)

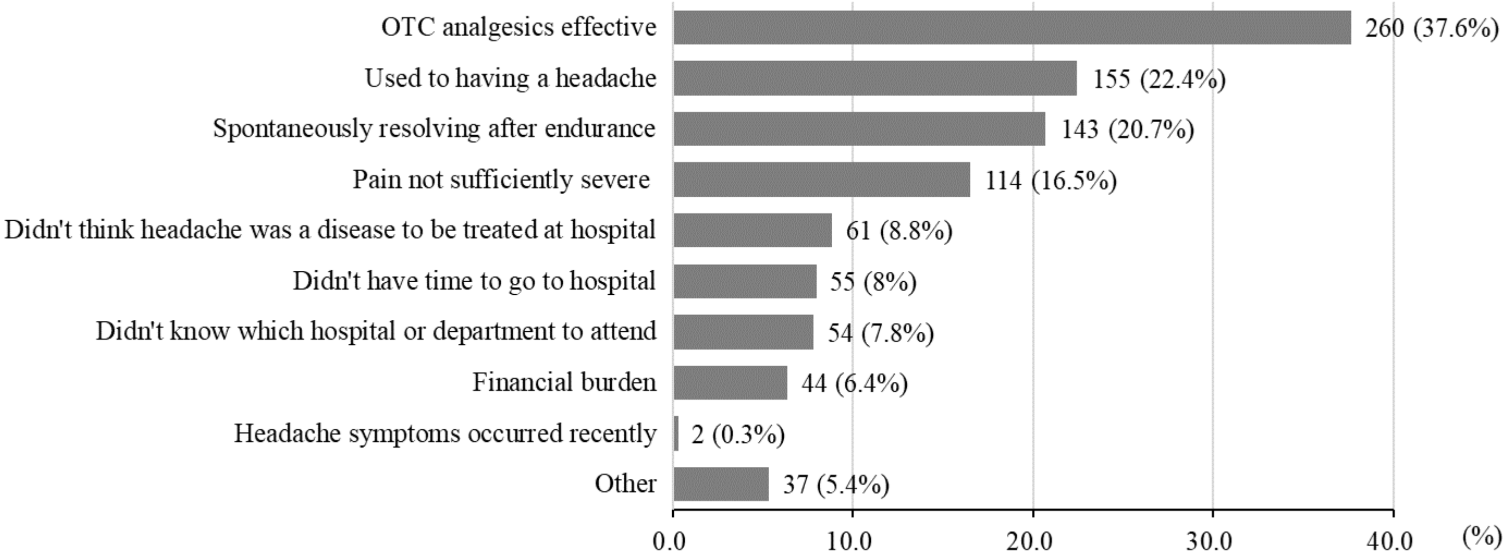

Abbreviations: OTC, over-the-counter.
