# Supplementary material for: A study to investigate the prevalence of headache disorders and migraine among people registered in a health insurance association in Japan
Source: J Headache Pain. 2022 Jun 23;23(1):70. doi: 10.1186/s10194-022-01439-3 (PMC9219245; doi:10.1186/s10194-022-01439-3)
Supplement: Supplementary file 6 — Additional file 6. Symptoms and triggers of migraine (N=691) [file 10194_2022_1439_MOESM6_ESM.pdf]

Additional file 6 Symptoms and triggers of migraine (N=691)

a) Symptoms associated with headache (multiple answers)

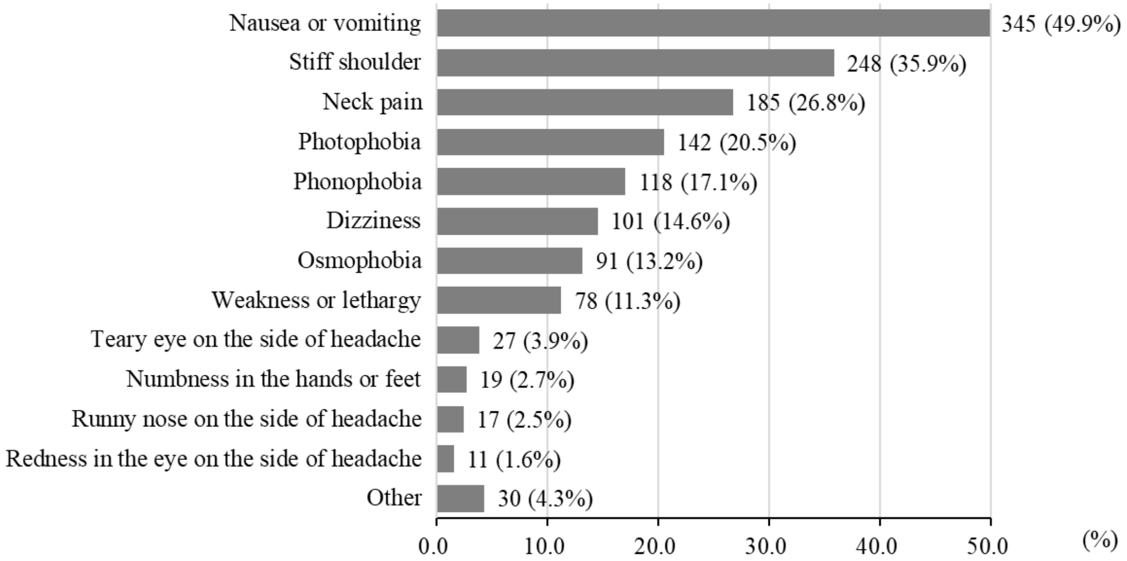

b) Headache triggers (multiple answers)

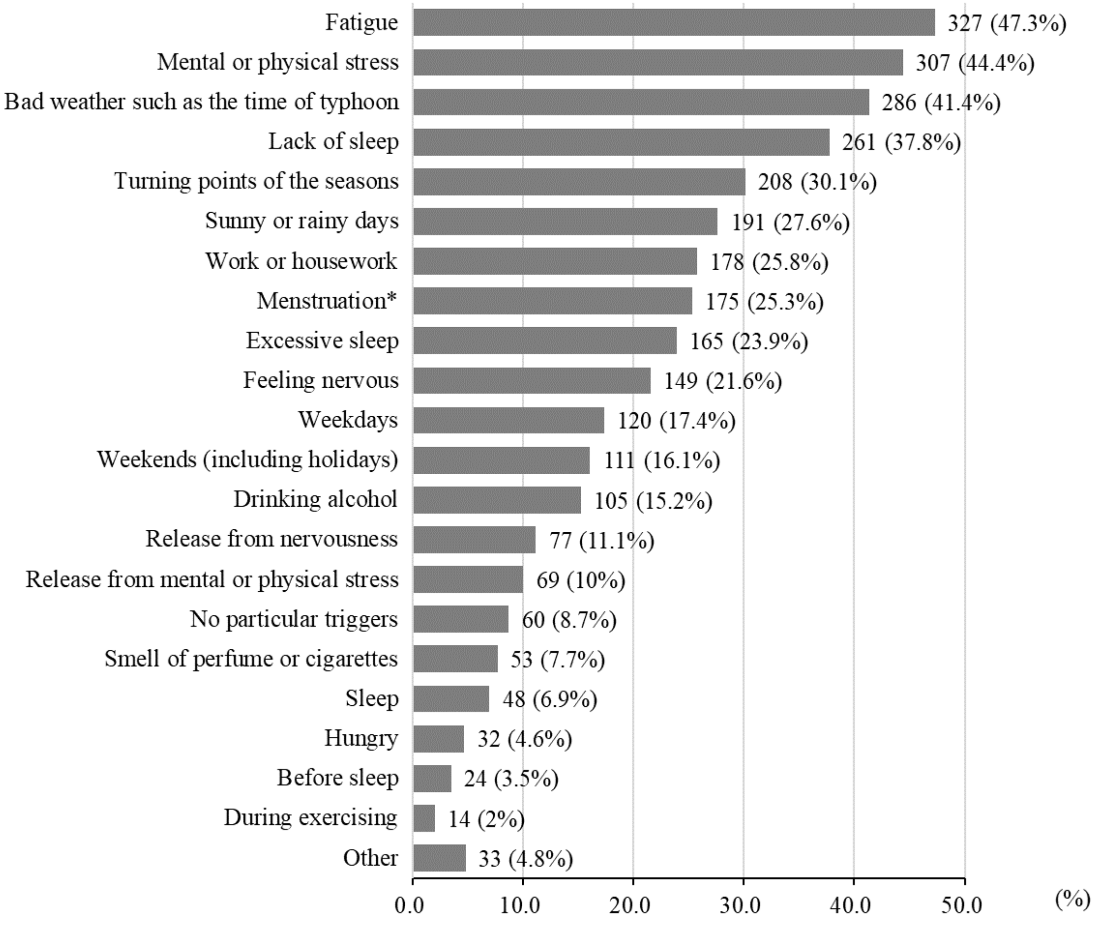

*Among those who had menstruation (325 patients), 171 patients (52.6%) answered "menstruation".
